# 

**Published:** 1893-11

**Authors:** 


					﻿AVOIDABLE DISEASES.
By C. B. NEWTON, M. D.
iq cts. a copv.
NOVEMBER, 1893.
$ i .00 per year.
HALL'S#
UOVRNALJ
HEALTH
ESTABLISHED 1854
Vol. XL.
No. 11.
OFFICE, 23 PARK ROW.
Entered as Second-Class Matter at the Post-Office, New York.
				

